# Concerns about Quadrupole ICP-MS Lead Isotopic Data and Interpretations in the Environment and Health Fields

**DOI:** 10.3390/ijerph15040723

**Published:** 2018-04-11

**Authors:** Brian Gulson, George D. Kamenov, William Manton, Michael Rabinowitz

**Affiliations:** 1 Energy and Environmental Research Centre, Department of Environmental Sciences, Macquarie University, Sydney, NSW 2109, Australia; 2 CSIRO Energy Flagship, North Ryde, NSW 2113, Australia; 3 Department of Geological Sciences, University of Florida, Gainesville, FL 32605, USA; kamenov@ufl.edu; 4 Department of Geosciences, University of Texas at Dallas, Richardson, TX 75080, USA; manton@utdallas.edu; 5 Marine Biological Laboratory, Woods Hole, MA 02543, USA; mrabinow@mbl.edu

**Keywords:** lead isotopes, ICP-MS, TIMS, MC-ICP-MS, environment, humans, rats, fractionation

## Abstract

There has been a massive increase in recent years of the use of lead (Pb) isotopes in attempts to better understand sources and pathways of Pb in the environment and in man or experimental animals. Unfortunately, there have been many cases where the quality of the isotopic data, especially that obtained by quadrupole inductively coupled plasma mass spectrometry (Q-ICP-MS), are questionable, resulting in questionable identification of potential sources, which, in turn, impacts study interpretation and conclusions. We present several cases where the isotopic data have compromised interpretation because of the use of only the major isotopes ^208^Pb/^206^Pb and ^207^Pb/^206^Pb, or their graphing in other combinations. We also present some examples comparing high precision data from thermal ionization (TIMS) or multi-collector plasma mass spectrometry (MC-ICP-MS) to illustrate the deficiency in the Q-ICP-MS data. In addition, we present cases where Pb isotopic ratios measured on Q-ICP-MS are virtually impossible for terrestrial samples. We also evaluate the Pb isotopic data for rat studies, which had concluded that Pb isotopic fractionation occurs between different organs and suggest that this notion of biological fractionation of Pb as an explanation for isotopic differences is not valid. Overall, the brief review of these case studies shows that Q-ICP-MS as commonly practiced is not a suitable technique for precise and accurate Pb isotopic analysis in the environment and health fields.

## 1. Introduction

Three main mass spectrometric methods have been used to obtain Pb isotopic data with high precision for thermal ionization (TIMS) and multi-collector plasma mass spectrometry (MC-ICP-MS) and medium to low precision with a single detector magnetic-sector field ICP-MS (ICP-SMS) and quadrupole Q-ICP-MS. Time of flight ICP-MS has occasionally been used [1]. In recent years MC-ICP-MS Pb analysis using thallium for mass-bias correction has become the method of choice for high-precision Pb isotope work [2,3]. TIMS and MC-ICP-MS are instruments with multiple detectors designed specifically for isotopic ratio determination and are used in geochemistry where high precision and accuracy of isotopic ratios are necessary for interpretation. The disadvantages of these instruments are their high cost and the need for labor-intensive sample preparation in ultraclean laboratories. In contrast, the environmental and environmental health fields are dominated by isotopic measurements obtained with Q-ICP-MS because of the relative cheapness and minimal sample preparation. However, Q-ICP-MS are designed primarily for elemental concentration quantification based on external calibration. Due to the single detector configuration, Q-ICP-MS cannot simultaneously measure multiple isotopes as can TIMS and MC-ICP-MS. In recent years there has been an explosion in the number of papers reporting Pb isotopic data with researchers recognizing the possibility of source/pathway discrimination to resolve environmental and health problems. Unfortunately, there have been many cases where the quality of the isotopic data obtained by Q-ICP-MS are questionable with consequent impacts on interpretation and conclusions [4].

In addition to using the appropriate instrumentation, the successful use of Pb isotopes in source apportionment for environmental and biologic samples demands that there is a significant difference between the isotopic composition of the target media and the sources [5,6,7,8]. For example, in the Pearl River Delta region of southeastern China, after phasing out of leaded petrol which had uniquely low Pb isotopic compositions, it has become almost impossible to distinguish Pb isotopic compositions of urban dusts from those of the potential sources including coal, which is one of the main sources of lead contamination in China. In areas like southeast China where Pb isotopic compositions of the natural and anthropogenic sources including regional Pb-ore deposits are similar and highly radiogenic (e.g., high ^206^Pb/^204^Pb ratios) source differentiation of Pb contamination should not be based solely on Pb isotopic ratios [6]. Similarly, in one vacant US house, Gwiazda and Smith [7] concluded that isotopic ratios alone can lead to an overestimation as well as an underestimation of the true source of the Pb pollution in the environment, especially if the sample isotopic ratio represents a composite of Pb from different radiogenic sources. A comprehensive database is also necessary of Pb isotopic compositions of all traditional (such as coal and Pb-ores) and non-traditional potential sources including but not limited to crude-oil, unleaded petrol, diesel fuel, marine engine fuels and other industrial activities [6]. This is a difficult task as determined in studies which sought to observe Pb isotopic ratio patterns by brands of paint or refined metal ore where there was considerable variation and overlap in the isotopic ratios [9,10]. Establishing a high-quality database, and thus relevant constraints, is important, especially where there are several different potential sources and their relative contributions may vary significantly depending on the site-specific conditions.

Furthermore, it is important to measure all 4 isotopes of Pb [11]: ^208^Pb derived from radioactive decay of ^232^Th, ^207^Pb from ^235^U, ^206^Pb from ^238^U and the primordial ^204^Pb which has no radioactive parent and whose abundance has not changed over geologic time. The low abundance of ^204^Pb of <2% limits precise measurements by Q-ICP-MS and hence only data for the 3 major isotopes are generally reported. Unfortunately, the omission of ^204^Pb may limit source characterization which is only obvious with ^204^Pb-based isotopic data [11], as will be seen in this paper. In addition, ^204^Hg, often present in Ar gas used in ICP-MS, also may impede precise determination of ^204^Pb although a Hg trap may be incorporated into the Ar gas line. Furthermore, during MC-ICP-MS Pb isotopic analyses ^202^Hg is routinely measured to correct for potential ^204^Hg isobaric interference on ^204^Pb [3,12]. If the isotopic data are not accurate or imprecise then any source characterization will be severely minimized or virtually negated. Interpretations using only 3 Pb isotopes may be valid when simple mixing of two components with widely differing ratios for the source materials provides data which plot along a linear array but this is rarely the case in real life where there can be multiple sources (see Section 3). In many cases, authors will state there are numerous multiple sources to explain their data but provide minimal supporting data for the sources to justify the claim. Besides the lower precision of Q-ICP-MS measurements, the minimal sample preparation can result in interferences or mass bias arising from matrix effects and resulting in unrealistic data, especially for organic/biological samples, which may not be obvious to the researcher, as discussed in this paper. Whilst on the subject of mass bias and validity of data in Q-ICP-MS measurements, many authors report the precision of their data based on replicate measurements of pure Pb standards such as NBS SRM981 (“common lead”), rather than replicate analyses (including digestion) of materials of similar matrix to the samples being analyzed. However, the SRM 981 measurements are for correcting mass bias, not precision so this is an unrealistic estimation of the precision and accuracy of their data. SRM 981 can be used for precision estimate only if Pb was purified from the samples on ion exchange columns, which is typically not the case for Q-ICP-MS studies [2].

We present several case studies which illustrate deficiencies or complications in isotopic data which have resulted in less than satisfactory interpretations of source apportionment. We provide examples where ICP-MS measurements on the same samples have also been made with MC-ICP-MS or TIMS after Pb purification on ion-exchange columns. In addition, we also provide examples where precise isotopic data implementing the ^204^Pb-based ratios allow for refined interpretations not feasible with only the major isotopic ratios of, for example, ^208^Pb/^206^Pb versus ^207^Pb/^206^Pb. We focus this Commentary mainly on studies which involve organic or biological samples.

## 2. Methods

This is not intended as a comprehensive review of Pb isotopic investigations in the environment [13] and health fields [14,15] but to present some examples where Q-ICP-MS data have deficiencies, examples with higher quality ICP-SMS data and finally some TIMS studies.

There are three major requirements for accurate and high-precision Pb isotopic analysis. First, all of the sample preparation must be performed under clean laboratory environments using ultra-pure reagents. This step is required to control the Pb blank so that the blank to sample levels have negligible impact on the measured ratios. Second, Pb must be isolated from the sample matrix using ion-exchange resin. Typically this is done in HBr medium using Dowex 1X-8 resin (Sigma-Aldrich, St. Louis, MO, USA) or Pb ion selective resin. This step is required to eliminate the so-called matrix effects, which affect the ionization, transmission, and interferences of analyte elements. In ICP-MS in particular, matrix effects cause spectral and non-spectral interferences and have detrimental effects on the precision and accuracy of isotopic measurements [2]. Third, the purified Pb fraction must be analyzed either on MC-ICP-MS, using Tl or double-spike (DS) or on TIMS, preferably also using DS if ultra-high precision is required, not always a condition in many environmental studies [2]. Multi-collector configuration of both instruments allows for simultaneous acquisition of all of the isotopes of interest, including double-spike Pb or Tl isotopes, required for accurate mass-bias correction.

In addition, both TIMS and MC-ICP-MS utilize Faraday detectors (the designated choice for high-precision isotope work), in contrast to single-collector ICP-MS, which are typically fitted with single secondary electron multiplier (SEM) detector. SEM detectors have inferior long-term stability, compared with Faraday detectors and also suffer from other issues like dead time. The dead time results in missing counts at high ion beam intensities can be detrimental when SEM detectors are used to measure ratios for elements with very different isotopic abundances. At present, high-precision Pb isotopes can only be obtained after ion-exchange Pb purification and by analysis on MC-ICP-MS and/or TIMS with Tl or double-spike mass-bias correction providing isotope precisions of <0.01% RSD for ratios approaching unity [16].

### How Can One Check the Data and Evaluation of Sources?

Although the 3 main mass spectrometer systems have issues with mass bias and isobaric interference, this is more an issue with Q-ICP-MS because of the plasma sample introduction and quadrupole detection systems. As mentioned above, to correct for mass bias, most researchers measure a pure metal standard such as SRM 981 and very commonly those measuring isotopic compositions with Q-ICP-MS report these data as a guide for the precision of their measurements. However, this is not a true indication of the precision of real samples as this can only be achieved by replicate measurements of samples of similar composition to those being investigated. In many studies, samples are simply digested and then introduced into the mass spectrometer resulting in mass bias and interference. To minimize these problems and obtain higher quality data, some researchers undertake Pb purification—as necessitated for TIMS and MC-ICP-MS measurements—involving ion exchange methods [15,17] and digest samples using microwave techniques [18] although the latter can result in high levels of dissolved organic carbon which lead to matrix effects. Once the isotopic compositions have been measured the accuracy of the data can be checked by graphing. In most cases in environmental and health literature this is from 3 isotope ratio plots (e.g., ^208^Pb/^206^Pb versus ^207^Pb/^206^Pb, the inverse or a combination of these). By convention in the geological field and to display maximized dispersion, the ^206^Pb/^204^Pb, ^206^Pb/^208^Pb (or inverse) and ^206^Pb/^207^Pb (or inverse) are usually chosen to characterize potential sources or pools of Pb. Transforming the observed ratios into eigenvectors has yielded no superior resolution between sources [19]. If the researcher reports ^204^Pb-based ratios, this should also be checked, for example, by plots of ^207^Pb/^204^Pb versus ^206^Pb/^204^Pb or ^208^Pb/^204^Pb versus ^206^Pb/^204^Pb although this may cause consternation if the data exhibit a large scatter as is common with Q-ICP-MS results and the ratios are outside the range of know terrestrial isotopic compositions as discussed in the following section.

**Figure 1:**
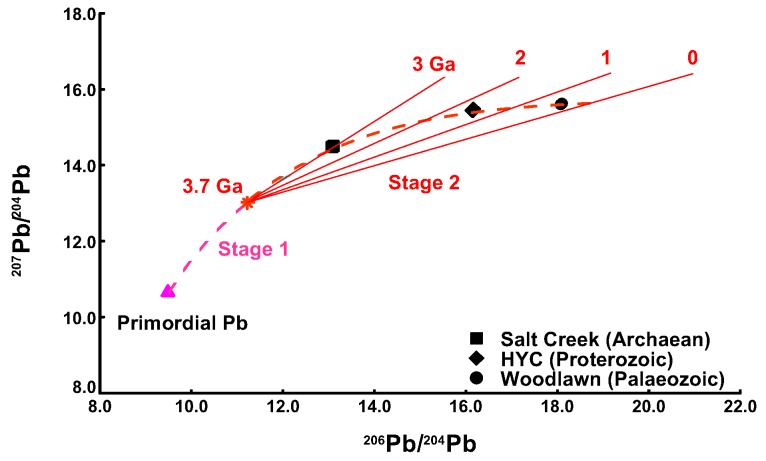
Stacey and Kramers (1975) two stage Pb evolution curve also showing Pb isotopic ratios for three high-Pb ore deposits of different geological ages (data from, [24]).

Evaluation of more than two Pb sources is difficult unless high-quality isotopic data are available and/or there are large differences in the isotopic composition of the sources. The bases for interpretation of Pb isotopic data relies on two major factors: (1) is the radioactive decay schemes of U to Pb and Th to Pb and (2) the relationship of the measured data to the Pb evolution curves (growth curves) for Pb-rich massive sulphide ore bodies [20,21]; (Figures 1 and 6, this paper). The basic equation for the ^206^Pb/^204^Pb is:

^206^Pb/^204^Pb = ^206^Pb/^204^Pb_i_ + ^238^U/^204^Pb (e^λt^ − 1),

where ^238^U/^204^Pb is the source rock U/Pb, ^206^Pb/^204^Pb_i_ is the initial ^206^Pb/^204^Pb_,_ λ is the decay constant of ^238^U and t is time.

In the case of the first point, the minimum (initial) ^206^Pb/^204^Pb in terrestrial samples is about 9.3, (Primordial Pb in Figure 1) based on earlier measurements in iron meteorites by Patterson [22] as the first accurate determination of the age of the earth at about 4.55 billion years, so that any ^206^Pb/^204^Pb results less than 9 are unrealistic.

The measured data can also be plotted relative to Pb evolution curves and the isotopic ratios depend on the ore bodies from which they are derived (anthropogenic sources) and any influences due to radioactive decay (as occurs in natural samples such as rocks and soil). The growth curves mentioned above are the simplest form (single stage models) but more realistically there are more complex systematics (multi-stage models) especially if there are differences in the U/Pb ratio in the source regions (e.g., Stacey and Kramers [21] model, illustrated in Figure 1).Thus in the isotopic ratio plots mentioned above, the natural and anthropogenic sources are considered to be end-members of a mixing model and the investigated samples should plot in an area which can be delineated by the potential source(s). In an ideal case, the measured samples may be expected to define a linear trend reflecting a binary mixing model. Ellam [11] provided a lucid explanation on plotting of Pb isotopic data and concluded that a plot of ^206^Pb/^208^Pb and ^206^Pb/^207^Pb was not a suitable diagram to reveal multiples sources of Pb as most terrestrial Pb data are co-linear in the diagram; it will always yield the same linear trend irrespective of the number of sources of Pb. Likewise, he suggests that a plot of ^206^Pb/^204^Pb versus ^206^Pb/^207^Pb, which is occasionally used in environmental studies (e.g., [23]), is inappropriate with which to identify multiple sources of terrestrial Pb and any number of mixing sources will yield a correlation that could be misinterpreted as reflecting binary mixing.

## 3. Results

### 3.1. Examples Using Organic Extractions and Different ICP Instruments

To obtain more information about accessibility of metals, some researchers have employed EDTA extraction, a method commonly used in soil science and compared these results with acid digestions.

**Figure 2:**
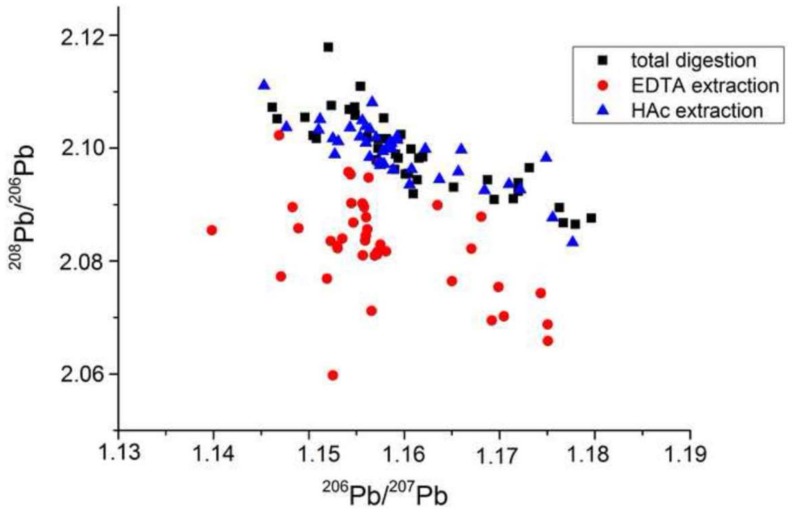
Isotopic compositions in digests and extracts from urban soils of Athens showing the scatter of data for the EDTA extractions probably arising from matrix effects during inductively coupled plasma-mass spectrometry (Q-ICP-MS) analyses compared with the expected linear arrays for the total digestions and HAc extractions. (From [25]).

For example, Kelepertzis et al. [25] extracted metals from Athens (Greece) urban soils and house dust from Athens using total digestions (HNO_3_, HCl, HF), EDTA and acetic acid (HAc). The isotopic composition of ^206^Pb, ^207^Pb and ^208^Pb was determined using Q-ICP-MS (ICapQ, Thermo Scientific, Germany). Correction for mass bias during the determination of the isotopic ratios was performed using analyses of NIST SRM 981 after every two samples. The standard errors for measurement of the ^206^Pb/^207^Pb were <0.3% RSD and <0.4% RSD for ^208^Pb/^206^Pb ratios, assumed to be for SRM 981. The results, plotted as ^208^Pb/^206^Pb versus ^206^Pb/^207^Pb (Figure 2), for total digestions and acetic acid scatter about a linear array as might be expected at least for total digestions whereas those for EDTA extractions lie below the array and are inconsistent with data from terrestrial samples. Their reference material was the Montana Soil standard (NIST SRM 2711a). In contrast, Komarek et al. [26] extracted Pb from forest soil profiles sourced from a natural park, a smelting area and Prague motorway using EDTA, HNO_3_, aqua regia and total digestions (as above). The isotopic data scatter around a linear array, including those for the EDTA extraction. The accuracy of the Pb isotopic measurements was monitored by analysis of the standard AGV-2 (andesite), a satisfactory standard for total digestions. The measured ^206^Pb/^207^Pb ratio 1.2091 ± 0.0013 was in good agreement with the certified values of 1.2085 ± 0.0006. The difference between the two EDTA data sets could be explained by the difference in the ICP-MS. For example, that used by Kelepertzis et al. [25] was a bench model ICapQ whereas that used by Komarek et al. [26] was a PQExCell (VG) with collision cell (now Thermo). The use of the collision cell allows interfering elements to be combined with a variety of reaction gases and ‘mass shifted’, rather than being removed entirely [27]. An alternative reason for the Athens EDTA isotopic data may be from a change in mass bias due to the presence of EDTA in plasma (reviewers suggestion). No explanation for the unusual isotopic data for the Athens EDTA analyses was offered even though these were queried in a review of the manuscript by one of the authors of the current manuscript.

### 3.2. Other Complications

In a comprehensive investigation, blood from 100 adults and 56 children in 2012 were compared with 169 environmental samples (tap water, vacuum cleaner dust, paint, country food, soil and ammunition) collected from 14 houses from three Inuit communities in northern Canada. Subjects were those identified during the Inuit Health Survey of 2008–2009 who had the highest blood Pb levels [28]. Total Pb levels and Pb isotope mass balance were determined by inductively coupled plasma-mass spectrometry (Q-ICP-MS) using an Agilent ICP-MS (7700 Series) fitted with a MicroMist nebulizer and a quartz spray chamber. Certified standard reference materials were used for QA/QC of Pb concentrations and accuracy/precision of isotopic analysis. For blood Pb, NIST Standard Reference Material 955c (levels 1–4) was used. Reference materials provided by the GhostWipe™ manufacturer (Environmental Express, Charleston, SC, USA) were used for QA/QC. For house dust, paint, soil and ammunition, Buffalo River Sediments reference material (NIST #8704) was used for QA/QC. For food samples, fish protein reference material (NRC DORM 3) was used for QA/QC. Blanks and check standards were run every ten samples. Recovery rates of all standard reference materials were between 93% and 110%. Unfortunately, no Pb isotopic data were reported for any of the reference materials.

In a novel approach [28], the relative isotopic composition of blood and environmental samples was investigated in households where at least one participant had a blood Pb level higher than the earlier CDC guideline of 10 μg/dL, using discriminant analysis. This method is different from the usual comparison of isotopic ratios in graphs, as it takes into account all four stable isotopes of Pb simultaneously and attempts to model the differences between classes of data and recognize patterns. Discriminant analyses and isotopic ratios analyses showed that ammunition and house dust were the major sources of Pb in this study population.

**Figure 3:**
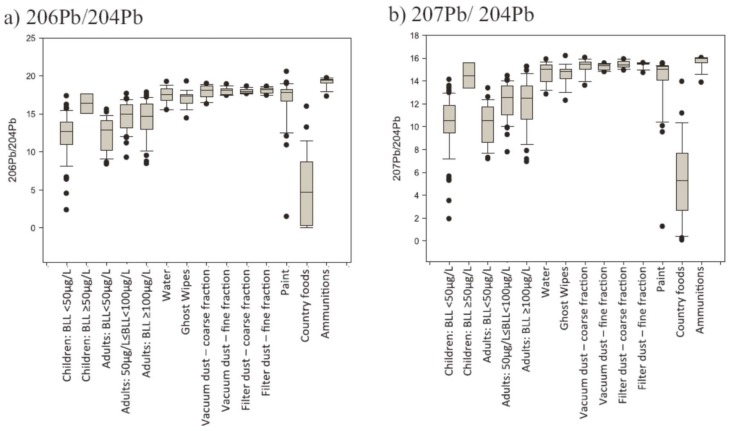
Isotopic ratios in blood and environmental samples from the study of Fillion et al. [28] showing some impossible low ^204^Pb-based ratios for terrestrial samples such as some blood samples and the country foods.

However, consideration of the isotopic data shown in Figure 3, especially for the low ^204^Pb-based ratios, shows impossible Pb values. Such Pb isotopic ratios would be considered ‘extraterrestrial’ or even “extra-solar system” (e.g., ^206^Pb/^204^Pb < 6, Figure 3a), that is, they have not been observed in any earth, lunar, or meteorite samples so far analyzed.

In evaluating the source of Pb in Mute swans wintering in northern Poland, Binkowski et al. [29] measured isotopic ratios in blood and ammunition pellets with an Agilent 7700 series ICP-MS. The authors carried out analyses of reagent blanks, fully repeated and parallel samples, as well as the standard SRM 981 and, although they claimed the results were satisfactory, none of these data were documented.

**Figure 4:**
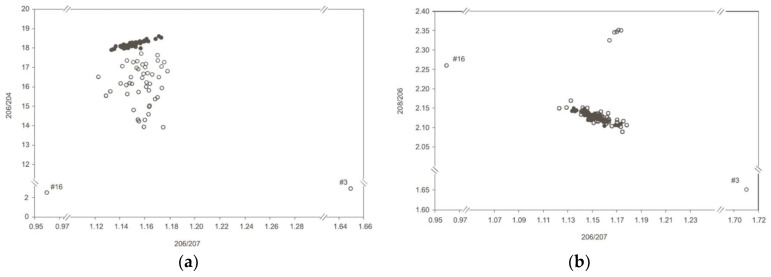
(**a**) ^206^Pb/^204^Pb versus ^206^Pb/^207^Pb plot of blood (open circles) from swans and ammunition pellets showing the large scatter of data possibly arising from analytical problems for the blood samples. (**b**). ^208^Pb/^206^Pb versus ^206^Pb/^207^Pb plot showing consistency in blood and pellet data for most samples. (Data from [29]).

The data for the pellets lay on a fairly well-defined mixing line but those for the blood scattered widely on a ^206^Pb/^204^Pb versus ^206^Pb/^207^Pb plot and especially 2 outliers with impossible ratios for terrestrial samples (Figure 4a). On a ^208^Pb/^206^Pb versus ^206^Pb/^207^Pb plot most of the blood and pellet data show good overlap but 7 blood samples were outliers. The authors attributed the outliers to exposure of these birds to different sources of Pb compared with the other birds rather than a possible analytical artifact.

### 3.3. Comparison of ICP-MS and TIMS/MC-ICP-MS

**Figure 5:**
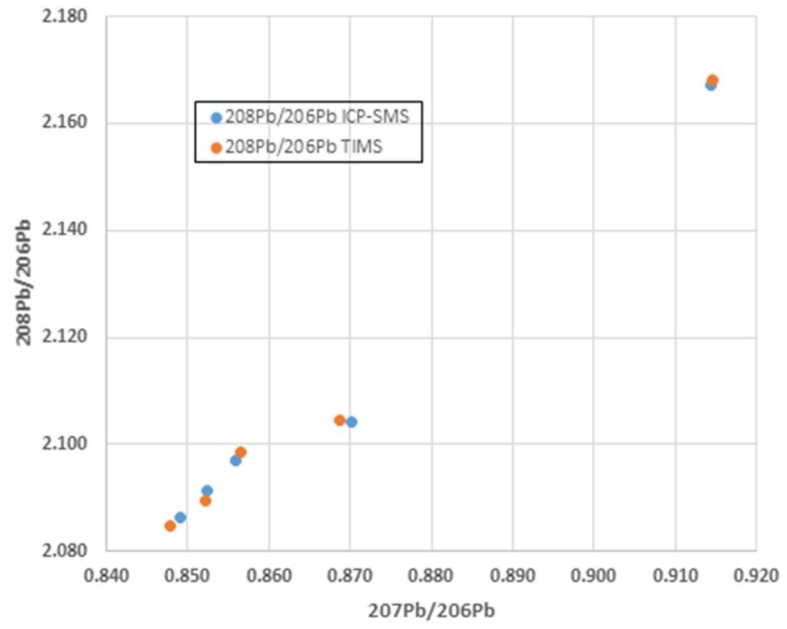
Excellent agreement in major isotopic ratios between thermal ionization (TIMS) and single detector magnetic-sector field ICP-MS (ICP-SMS) associated with rigorous sample preparation and analysis (data from [30]).

There have been several investigations comparing sector-field ICP-MS and TIMS/MC-ICP-MS Pb isotopic data which have shown that with much care, especially in sample preparation (e.g., microwave digestion/strong acids such as HF to minimize matrix effects) and rigorous monitoring it is possible to obtain precisions of ±0.05% in ^207^Pb/^206^Pb and ±0.1% in ^208^Pb/^206^Pb ratios for samples with Pb amounts in the range of 1 mg and 0.1 mg on single collector ICP-SMS (e.g., [30]). In general, the Pb isotope ratios determined using ICP-SMS deviated from the TIMS values by less than 0.1% (Figure 5). All ICP-MS measurements were carried out with an Element 2 ICP-SMS (Thermo Finnigan, Bremen, Germany) equipped with a guard electrode to eliminate secondary discharge in the plasma and to enhance overall sensitivity. The high-resolution double focusing (reverse Nier–Johnson geometry) single collector ICP-SMS instrument provides flat top peaks in the low-resolution mode (m/Dm 300) which was used.

**Figure 6:**
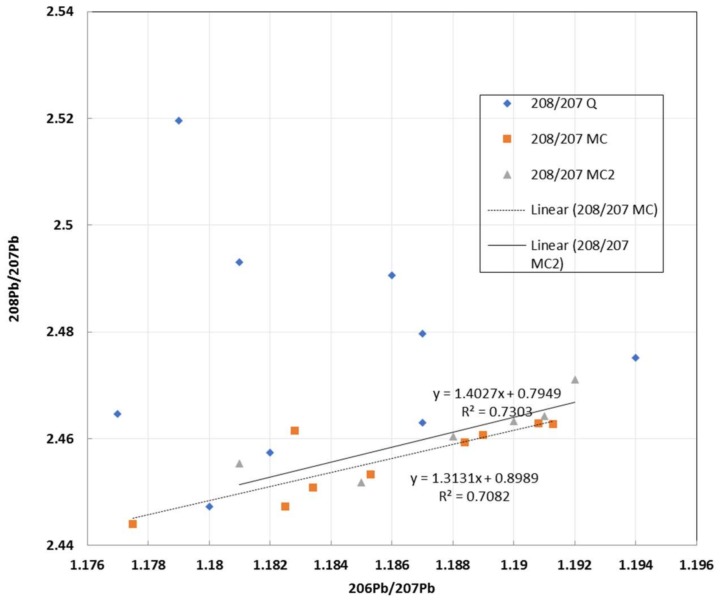
Comparison of Q-ICP-MS and multi-collector plasma mass spectrometry (MC-ICP-MS) data for peat bogs from Canada and Scotland (data from [31]) showing the lack of discrimination for the earlier Q-ICP-MS data. MC 1 and 2 data are for different depths.

In an investigation of depth profiles from peat bogs in Scotland (site LL7c) and eastern Canada (site PeW1), Kylander et al. [31] compared MC-ICP-MS data with the quadrupole ICP-MS data obtained earlier from 2002 Weiss et al. [32]. Q-ICP-MS data, even without using ^204^Pb, are highly inaccurate and show large scatter when compared with the MC-ICP-MS data (Figure 6).

Furthermore, deficient interpretations in using plots of major isotopic ratios compared with ^204^Pb-based ratios, were demonstrated by Weis et al. [33] investigating atmospheric Pb deposition in 5 Swiss peat profiles. In plots of ^206^Pb/^207^Pb versus ^206^Pb/^204^Pb the ratios measured by high precision TIMS defined a mixing line linking two possible end members (their Figure 8) and they concluded that such a linear array did not allow the source(s) of pollution to be defined unequivocally. However, in plotting the data as the ^204^Pb-based ratios (their Figure 8) they concluded that the simple binary interpretation was overly simplistic.

### 3.4. Examples Where the Major Isotopes Fail

Below we present three examples where use of just the major isotopes alone does not allow elucidation of the different sources of Pb.

#### 3.4.1. Esperance Western Australia 

**Figure 7:**
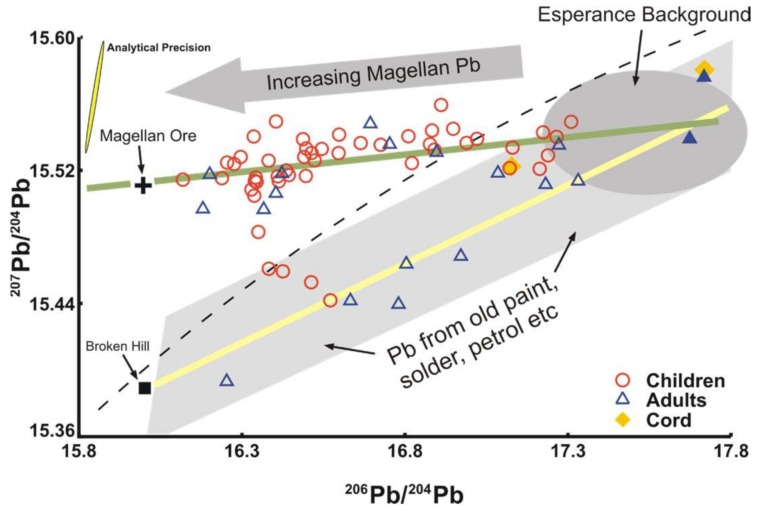
Conventional (^204^Pb-based) isotope ratio plot for uranium-derived Pb isotopes. The dashed line is the Growth Curve or Pb Evolution Curve along which most massive sulfide deposits lie, such as Broken Hill. The green line is the line of best fit for the isotope data termed the “Magellan Array” and the yellow line the “Old Array”, described in the text. The hatched fields denoted by arrows are estimations of the possible isotopic fingerprints. The data represented by blue filled symbols with the highest ^206^Pb/^204^Pb values could lie on either array. The 95% confidence ellipse in the upper left-hand corner represents the measuring precision.

In late 2006, the seaside community in Esperance Western Australia was alerted to thousands of native bird species dying [34,35]. The source of the lead (Pb) was thought to derive from the handling of Pb carbonate concentrate (Magellan mine) through the port, begun in July 2005. Concern was expressed for the impact of this on the community. Only plots of ^207^Pb/^204^Pb versus ^206^Pb/^204^Pb were able to discriminate the source of the Pb because of the almost identical ^206^Pb/^204^Pb ratio of the concentrate and that of the major Broken Hill Ag-Pb-Zn ores, the predominant anthropogenic source of Pb in Australia (Figure 7). Several sets of samples were specifically analyzed for use in the legal proceedings against the Esperance Port Authority and the mining company. These included the ore Pb concentrate, the small suite of blood samples from 7 children whose PbB ranged from 3.3 to 18.9 µg/dL, sediments from the harbor, soil samples, bird livers and rainwater tank samples. The overwhelming component of Pb in these samples was from a Magellan mine source [34,35].

The isotopic data for blood samples lay around two distinct arrays (Figure 7). The high precision isotopic data were critical in the legal proceedings against the mining company as stated by the senior Western Australian Department of Environment and Conservation investigator [35]:

“…the Pb isotope work was pivotal in the case because without it, we would have had some difficulty in positively identifying the Esperance Port as the true offender” (Cameron Oxford, senior investigator, written communication, 22 September 2010). The legal proceedings have now been settled with the Esperance Port Authority being fined $525,000, the largest in Western Australia for pollution. The mining company agreed to provide $9 million over three years to clean up the town and another $1 million for an Esperance community fund (The West Australian newspaper, 30 October 2009).

#### 3.4.2. Ryde Bridge

**Figure 8:**
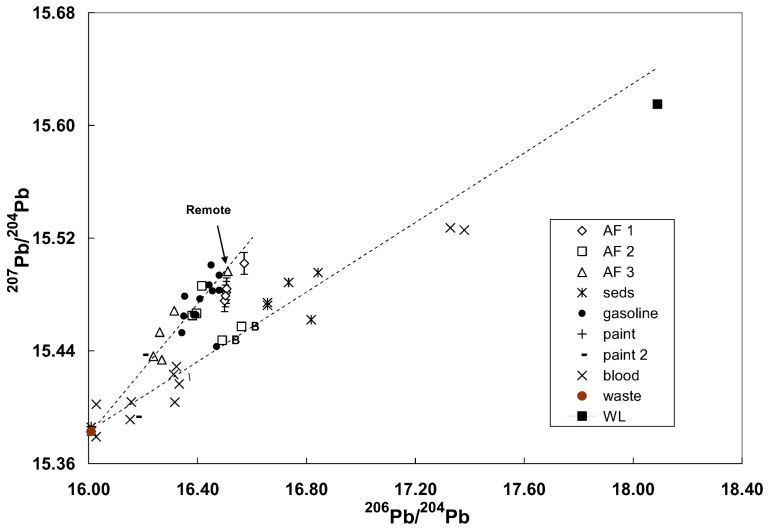
Conventional ^204^Pb-based isotope ratio plot for samples from the monitoring program. AF1 to AF3 refer to the 3 air filter sampling periods with AF3 being associated with possible release during abrasive blasting (waste). “B” denotes site B for the second air sampling program. The data for paint 1 and waste are the average values. Error bars are shown for air filter particulates from the first background sampling. The dashed lines are “eye-ball” fit to the data. Seds denote the underwater sediments collected under the bridge; WL are the values for a Pb-Zn mine about 400 million years old about 200 km south west of Sydney. The waste values are identical with the geologically ancient (~1700 million years old) Broken Hill mines.

**Figure 9:**
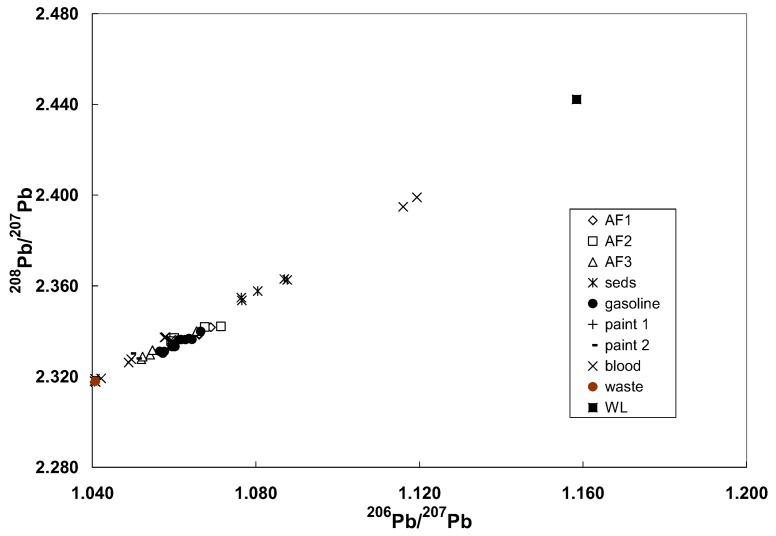
Plot of the isotopic data from Figure 7 on a ^208^Pb/^207^Pb versus ^206^Pb/^207^Pb diagram, commonly used in environmental investigations, showing the lack of discrimination using such a plot.

High precision Pb isotopic tracing was used for a bridge undergoing Pb paint removal to determine if Pb in the environmental and blood samples originated from the bridge paint [36]. Analysis of the red Pb primer gave uniform isotopic ratios indicative of Pb from the geologically-ancient Broken Hill mines in western New South Wales, Australia. Likewise, waste abrasive material, as anticipated, had the same isotopic composition as the paint. The isotopic ratios for other samples lay on 2 separate linear arrays on a ^207^Pb/^204^Pb versus ^206^Pb/^204^Pb diagram, one largely defined by gasoline and the majority of the ambient air data and the other by data for one sample each of gasoline and ambient air and underwater sediments (Figure 8). Isotopic ratios in background ambient air samples for the project were characteristic of leaded gasoline. Air sampling during paint removal showed a contribution of paint Pb ranging from about 20 to 40%. Isotopic ratios in the blood of 8 employees prior to the commencement of work showed that 6 of these had been previously exposed to the Broken Hill Pb possibly from earlier bridge paint removal projects. One subject appeared to have increased exposure to Pb probably from the paint renovations. A plot using the major isotopic ratios of ^208^Pb/^207^Pb versus ^206^Pb/^207^ could not discriminate between sources (Figure 9). It should be noted that many samples such as paint and soils contain high concentrations of Pb so that strong signals can be obtained allowing for reliable ^204^Pb measurements even using Q-ICP-MS.

#### 3.4.3. Atmospheric Aerosols 

**Figure 10:**
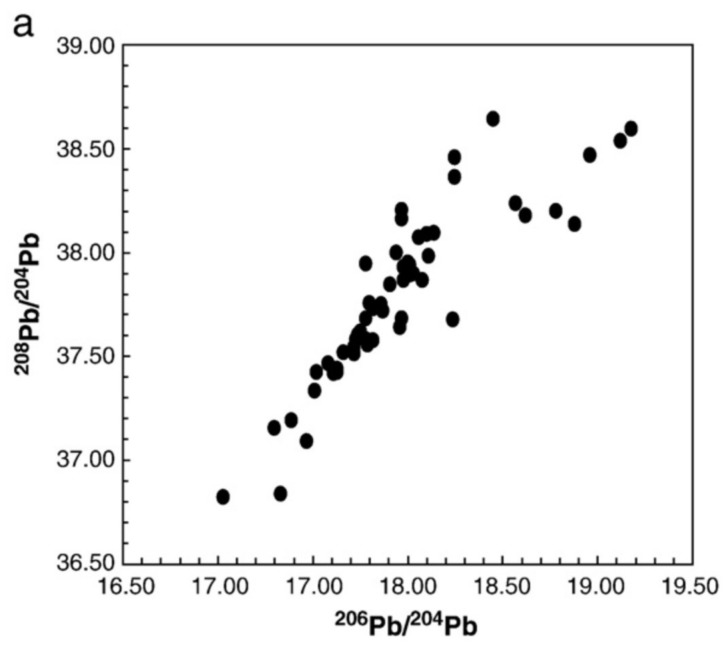

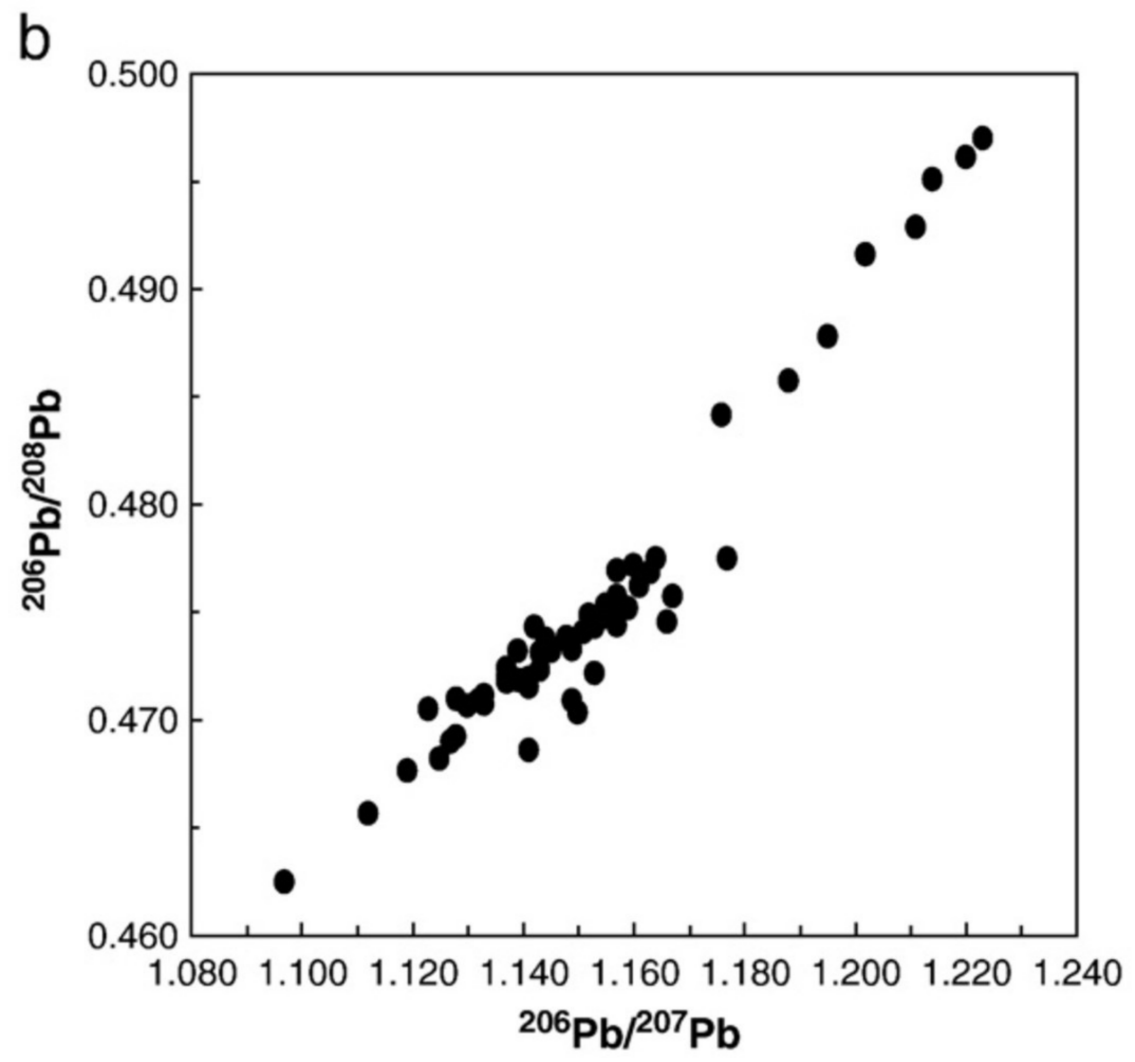
(**a**) ^208^Pb/^204^Pb versus ^206^Pb/^204^Pb and (**b**) ^206^Pb/^208^Pb versus ^206^Pb/^207^Pb for Northern Hemisphere atmospheric aerosols [37]. Only plotted are data for which individual analyses were presented by Bollhöfer and Rosman [37]. Sites for which ranges of Pb isotope composition were presented are omitted to avoid the possibility of plotting pairs of ratios that do not correspond to the same sample. (From [11]).

To illustrate the deficiencies in only plotting the major isotopes, Ellam [11] replotted the aerosol data of Bollhöfer and Rosman [37] on ^208^Pb/^204^Pb versus ^206^Pb/^204^Pb (Figure 10a) and ^206^Pb/^208^Pb and ^206^Pb/^207^Pb (Figure 10b) diagrams. “As identified by the original authors, these aerosols are likely to include Pb from a variety of sources. In ^208^Pb/^204^Pb versus ^206^Pb/^204^Pb space this is clearly indicated by a divergence from a linear correlation, especially for the more radiogenic samples. At least three distinct sources of Pb are indicated. However, in and ^206^Pb/^208^Pb and ^206^Pb/^207^Pb space a much stronger linear correlation is observed (r = 0.965). Notably, the samples with high ^206^Pb/^204^Pb for a given ^208^Pb/^204^Pb, that is, those with high ^206^Pb/^208^Pb (Figure 9a) are not readily identified as deviating from the overall best fit correlation in ^206^Pb/^208^Pb and ^206^Pb/^207^Pb space. Thus, without data for ^204^Pb, it would not be possible thoroughly to evaluate the sources of Pb contributing to these Northern Hemisphere aerosols.” (Ellam [11], (p. 3491).

### 3.5. Isotopic Fractionation in Biological Samples—Fact or Fiction?

Because of the high atomic weight of Pb and small differences between the isotopes, mass-dependent fractionation of the Pb isotopes has not been considered an issue, especially in environmental conditions. For example, strong oxidation processes involving sulphide ores such as galena produce materials in gossans and ironstones that retain the isotopic signature of the primary sulfides [38]. Lead isotopic fractionation can be neglected in most ancient metallurgical processes as the variations are so much smaller than the speculated values using the theoretical formula [39]. Similarly, Pb isotopic fractionation has not been discerned in biological systems involving humans. However, two studies on rats treated with Pb acetate purport to observe Pb isotopic fractionation because of exchange of Pb between different media. These results have been published by the same authors in 2012 by Wu et al. [40] and in 2014 by Liu et al. [41]. The 2012 studies provided results for blood, urine, and feces and, in addition to these, the 2014 paper gave results for kidneys, liver, lungs and bone. 

Both studies used identical protocols carried out in the same laboratories including the same analytical facilities. Six-week old male Sprague-Dawley rats in 3 experimental groups of 6 animals (low-dose, medium-dose and high-dose groups) were intratracheally instilled with 0.02, 0.2 and 2 mg/kg body weight of lead acetate (the “test” substance), respectively and compared with a control group.

When the rats received a continuous lead exposure, the whole PbB concentration increased correspondingly. The ^207^Pb/^206^Pb and ^208^Pb/^206^Pb ratios in blood were significantly negatively associated with whole-PbB levels for the low and medium dosing regimes. In contrast, at a PbB level of about 5 μg/dL for the high-dosed animals, the authors observed an inflection and until a PbB of 30 μg/dL, there was little change in ^207^Pb/^206^Pb and ^208^Pb/^206^Pb ratios. The authors [40] postulated that above 5 μg/dL, “…biological fractionation functions of the tissues became abnormal, which resulted in the differences in the isotope ratio between the blood and test substance to be reduced significantly”. This inflection appears to be similar to gradual saturation of red cell binding sites at high PbB levels i.e., PbB increases and then plateaus [42,43,44].

**Figure 11:**
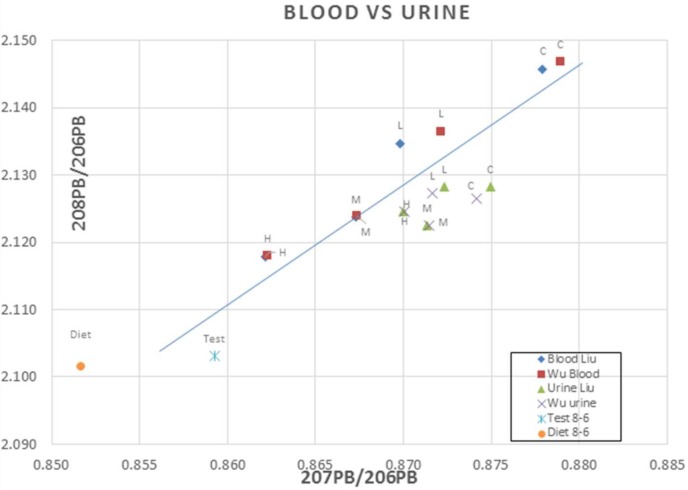
A comparison of ^207^Pb/^206^Pb and ^208^Pb/^206^Pb ratios in blood and urine from the two investigations. C denotes control, L low dose, M medium dose and H high dose.

There are several aspects of these papers which bring into question the conclusions of Pb isotopic fractionation:

(1) Digestion was with HNO_3_ and HCLO_4_ in quartz vessels but such equipment can contain small amounts of Pb and no processing blank data were provided.
(2) It is also unrealistic to quote Pb isotopic data to 5 decimal places, especially from Q-ICP-MS analyses, although the incredible agreement for blood and urine data from both studies shown in Figure 11 would permit such claims. It is also noteworthy that in the Wu et al. [40] paper the isotopic data are given as ratios but in the Liu et al. [41] the data are presented as % abundances of each isotope, although they do claim, to the contrary, that the % abundances are a better indicator of fractionation.
(3) There was no indication if the blood, urine and feces in Liu et al. [41] were the same data from the Wu et al. [40] paper although, as shown in Figure 10, the almost identical data for blood and urine from both studies and off-set of the urine data would perhaps indicate this. Such reproducibility for Q-ICP-MS is highly improbable.
(4) There are no Pb concentrations provided for the urine or any other organs.
(5) They state that Smith et al. [45] reported that there were large differences in the lead isotopic composition between paired blood and bone samples from each human subject. But this ignores the fact that the 5 subjects were from different countries, the long-term storage of Pb in bones and that 40–70% of Pb in blood under equilibrium conditions is derived from the skeleton [45,46,47,48,49,50].
(6) The authors appear to have ignored isotope systematics. Instead of fractionation, the linearity of the data for each dosing and control samples are examples of two-component mixing. Any tissue receiving inputs from blood and the “test” or diet will have isotopic data, expressed as ratios, that fall along a mixing line defined by the end-members and the linear distance along that mixing line will represent the relative contributions of each end-member to that tissue. Wu et al. [40] described such mixing but concluded that the isotopic differences in tissues were due to isotopic fractionation. Also, if we take the diet ^208^Pb/^206^Pb of about 2.10 and the blood and ^208^Pb/^206^Pb (highest close to 2.15) then 2.10/2.15 will be 2.4% fractionation. Such extraordinary fractionation is not even observed in light stable isotopes where fractionation is commonly found. Any variations in the isotopic makeup of the dietary inputs—which were not—as they slowly moved through the body were the drivers of the observed data, rather than fractionation.
(7) The rats were 6 weeks old so the control data should lie on same mixing line as with dietary data but, except for urine, all dosed data are offset from the Pb acetate “test” as in Figure 11.
(8) The bone data are inconsistent with the dosing regime (Supplementary Figure S1). For example, the medium bone data have lower ratios than the test data and the high dose bone data have ratios similar to the control and low dose samples.
(9) Data for blood/kidneys/liver/lung (Supplementary Figure S2) lie on linear trends between the diet and “test” but there are lower ratios in the low dose and medium dose livers that are inconsistent with the dosing regime. The lung data for all samples are identical to within experimental error which would be consistent with the intratracheal dose administration. Likewise, the kidney data for the dosed animals are almost identical to the lung data. For the high dose regime, the data for blood and other organs are the same to within experimental error which is what would be expected from biokinetics. (The higher uptake of Pb from diet or “test” in the liver for all animals is unexpected).
(10) The authors suggest that the ^204^Pb/^206^Pb ratio is a more sensitive biomarker in tracing Pb from inhalation but the errors for this ratio are much larger than for ^207^Pb/^206^Pb and ^208^Pb/^206^Pb ratios (Supplementary Figure S3).

In summary: there is no need to appeal to any thermodynamically limited, minuscule isotopic fractionation to explain the differences reported among their samples. Their data can be explained by the slow mixing of variable end members with Pb from the controls through the blood and other tissues. The inconsistencies in the Pb isotopic data for blood and soft tissues could be attributed to analytical problems due to usage of Q-ICP-MS.

### 3.6. Comparison with Other Rat Studies

The authors did not mention the seminal study of Smith et al. [51] on 8 female Wistar rats in which they used the highly sensitive approach of a stable isotope enriched in ^206^Pb in water, ingested by the rats (as against the intratracheal exposure by [40,41]). All processing of tissues (blood, kidney, vertebrae, tibia) was carried out in ultra-clean laboratories and the Pb isotopic ratios were measured by high precision TIMS. The isotopic ratios in the tissues differed by ~40% after 2 days of exposure to the ^206^Pb tracer. After 10 days exposure, more than 90% of the tracer isotopic signature was observed in the soft tissues (blood, kidney) whereas only ~50% was found in the skeletal tissues. Levels of the tracer in blood and kidney tissues varied between animals: in 3 of the treated animals, the levels were comparable whilst in the other 3 the levels were ≥10% higher in the kidney. On plots of ^208^Pb/^206^Pb versus ^207^Pb/^206^Pb for each animal, the data lay on tightly constrained linear arrays. Relationships between soft tissues observed by Smith et al. ([51] at days 4 and 6 are similar to those for blood and kidney found by Wu and Liu (see Supplementary Figure S2). Any variations in the uptake of tracer were attributed to inter-animal differences but not to isotopic fractionation.

### 3.7. Example of Laboratory Problem

**Figure 12:**
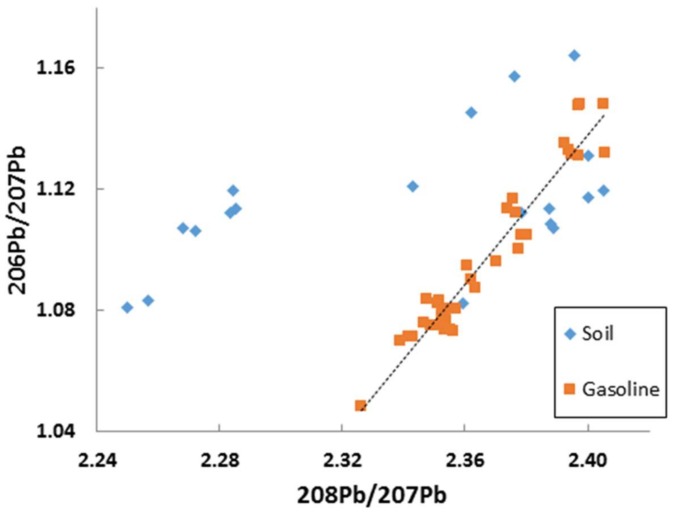
Major isotope ratio plot showing displacement of most of the soil data arising from analytical problems with ICP-MS

In spite of QA/QC protocols in place, especially in commercial laboratories, there are sometimes analytical problems giving rise to aberrant data even though by themselves the data appear valid. Such a case is shown in Figure 12 and because of the linearity of the soil data in the upper field, they could be misinterpreted as being realistic, especially by someone with limited experience in the use of Pb isotopes. Given the obvious differences in the arrays for the soils and the gasoline data [52], the researchers undertook a literature search and, in finding no similar data to the soils data in the upper field, re-analyzed the samples; the re-analyzed data were consistent with other data.

## 4. Conclusions

This brief literature review shows that Q-ICP-MS Pb isotope data can be highly erroneous and lead to false interpretations. Unless interpretations are straightforward, such as in the case of simple two-component mixing of end-members with a large isotopic spread, the veracity of at least some of the data should be checked with MC-ICP-MS or TIMS (e.g., as in the case of proposals for Pb isotopic fractionation in biological materials ([40,41]). Another deficiency in most Q-ICP-MS papers is lack of information on processing blanks, an issue when working with low Pb samples, especially biological ones and replicate data for real samples.

Acceptable quality ICP-MS data can be obtained with enhanced sample preparation and rigorous monitoring on double focusing single collector ICP-SMS in low-resolution mode (e.g., [30]). Rather than the expensive measuring systems such as MC-ICP-MS there have been developments in ICP-MS such as the tandem quadrupole ICP-MS. However, these single collector instruments are not likely to produce high precision isotopic analyses [27].

“For independent evaluation of published data by the scientific community, data sets from quality control measurements and experiments have to be accessible to the reader. This is usually not the case.” [2]. However, this is no longer an excuse with the inception of Supplementary Data files as standard for most journals.

Although researchers without access to MC-ICP-MS and/or TIMS may not be aware of the deficiency of Pb isotope data obtained with Q-ICP-MS, editors and reviewers must be aware of the problems with such analyses and should be responsible for the quality of the data and interpretations.

## Supplementary Materials

The following are available online at http://www.mdpi.com/1660-4601/15/4/723/s1, Figure S1: Comparison of ^207^Pb/^206^Pb and ^208^Pb/^206^Pb ratios in blood (Bl), urine (U) and bone showing significant off-set away from a mixing line for the control data and diet and the inconsistent values relative to the dosing regime for the bone data, including being slightly off-set from the “test” data, Figure S2: Comparison of ^207^Pb/^206^Pb and ^208^Pb/^206^Pb ratios in blood, liver, kidney and lung showing possible mixing lines with the lower section in between the diet and “test” data. The lower ratios in the low dose and medium dose livers are inconsistent with the dosing regime, Figure S3: Blood and urine data with error bars showing overlap between blood and urine for much of the data. The error bars for urine are similar to those for blood.
