# Supplementary material for: Concerns about Quadrupole ICP-MS Lead Isotopic Data and Interpretations in the Environment and Health Fields
Source: Int J Environ Res Public Health. 2018 Apr 11;15(4):723. doi: 10.3390/ijerph15040723 (PMC5923765; doi:10.3390/ijerph15040723)
Supplement: Supplementary file 1 [file ijerph-15-00723-s001.pdf]

International Journal of
Environmental Research and Public Health

MDPI

Supplementary Materials

Concerns about Quadrupole ICP-MS Lead Isotopic Data and Interpretations in the Environment and Health Fields

Brian Gulson, George D. Kamenov, William Manton and Michael Rabinowitz

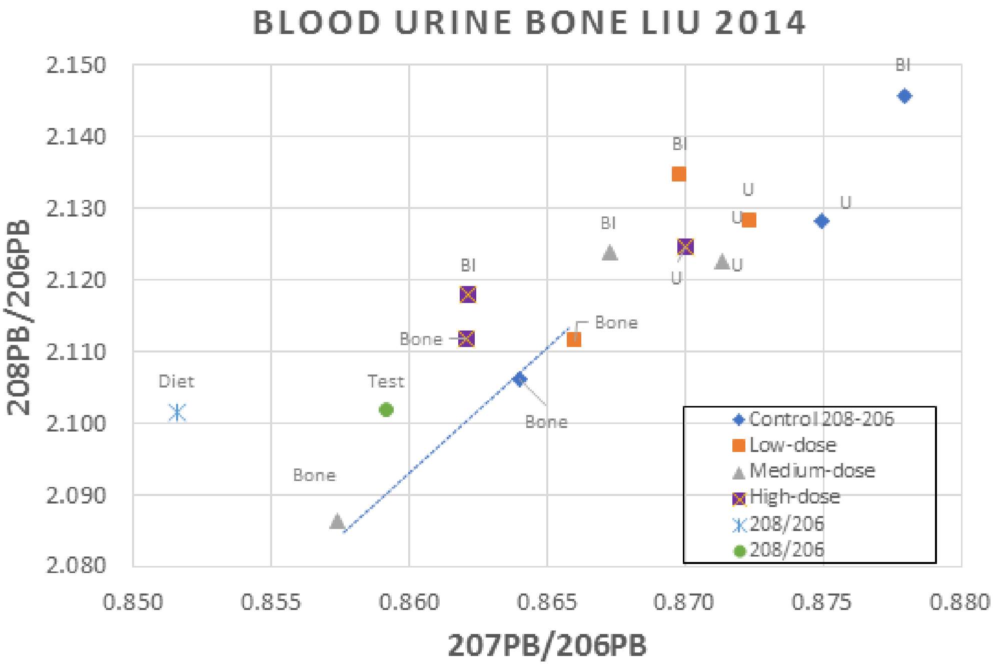

Figure S1. Comparison of $^{207}Pb/^{206}Pb$ and $^{208}Pb/^{206}Pb$ ratios in blood (Bl), urine (U) and bone showing significant off-set away from a mixing line for the control data and diet, and the inconsistent values relative to the dosing regime for the bone data, including being slightly off-set from the 'test' data.

International Journal of
Environmental Research and Public Health

MDPI

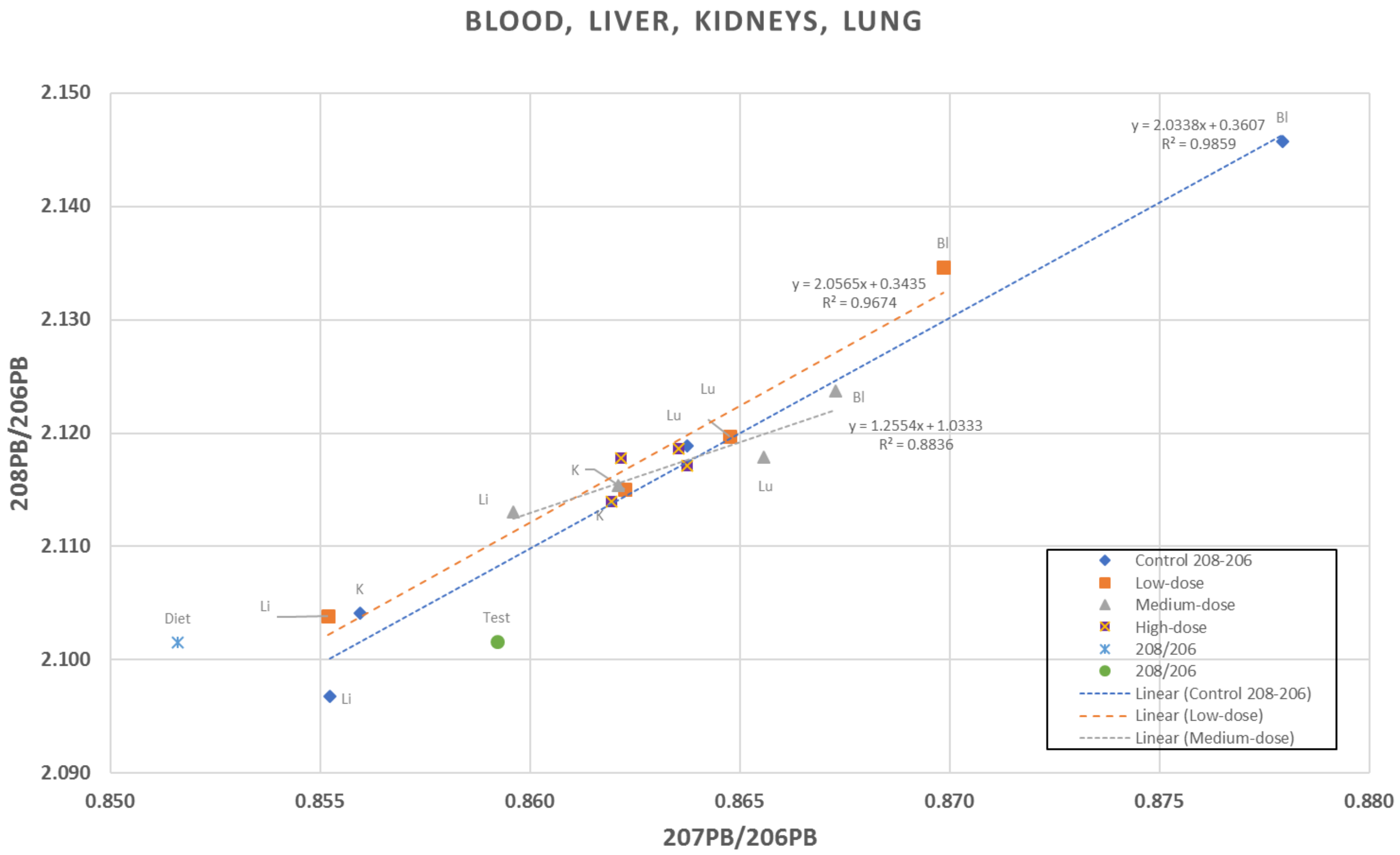

Figure S2. Comparison of $^{207}Pb/^{206}Pb$ and $^{208}Pb/^{206}Pb$ ratios in blood, liver, kidney, and lung showing possible mixing lines with the lower section in between the diet and 'test' data. The lower ratios in the low dose and medium dose livers are inconsistent with the dosing regime.

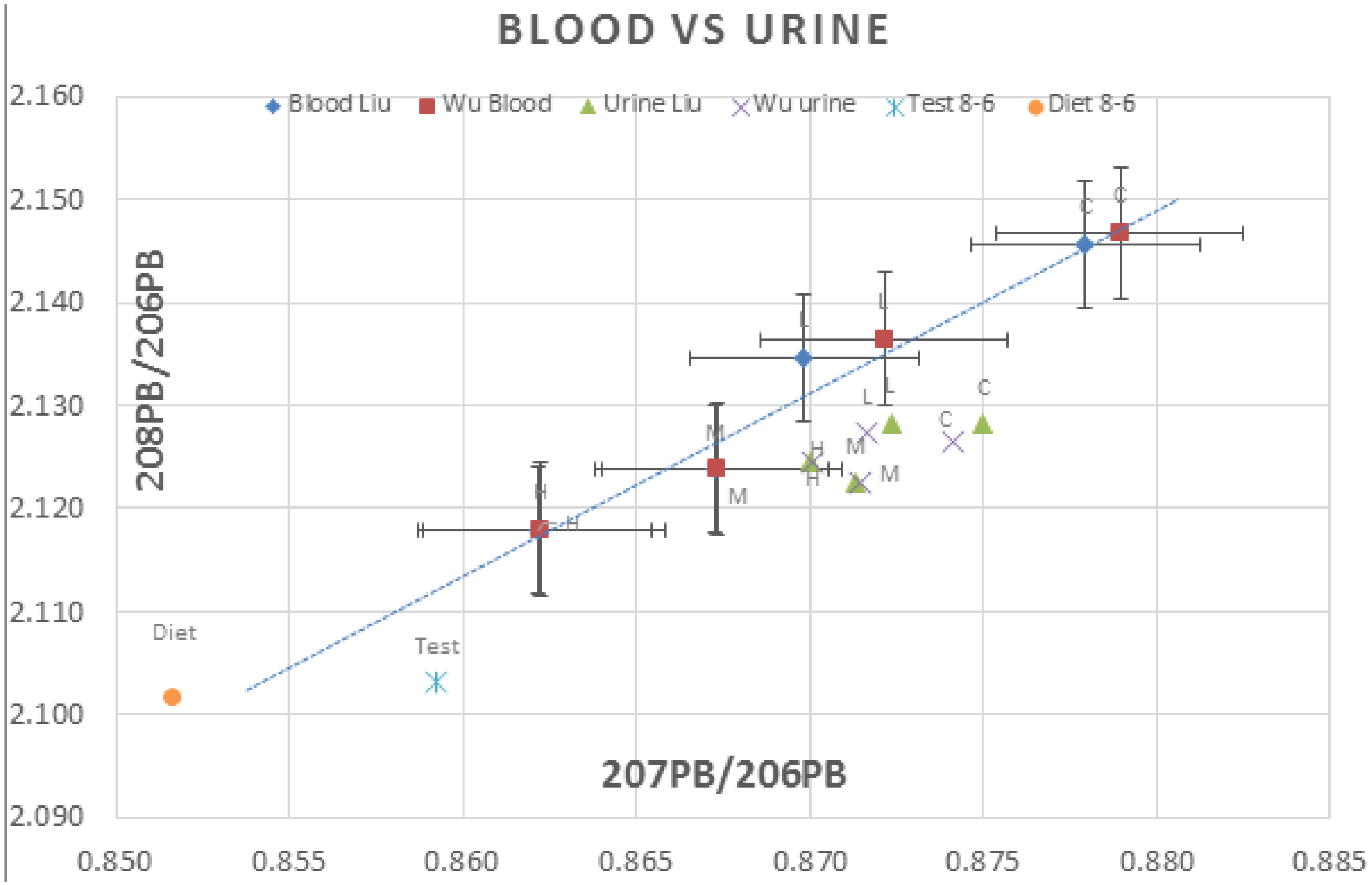

Figure S3. Blood and urine data with error bars showing overlap between blood and urine for much of the data. The error bars for urine are similar to those for blood.
